# Frontal Alpha Asymmetry and Electrodermal Activity: A Mutual Information Analysis Across Cognitive Load and Sleep Deprivation

**DOI:** 10.3390/bios16030164

**Published:** 2026-03-15

**Authors:** David Alejandro Martínez Vásquez, Hugo F. Posada-Quintero, Diego Mauricio Rivera Pinzón

**Affiliations:** 1 Department of Technology, Universidad Pedagógica Nacional, Bogotá 110221, Colombia; dmrivera@pedagogica.edu.co; 2 Electronic Engineering Faculty, Universidad Santo Tomás, Bogotá 110231, Colombia; 3 Department of Biomedical Engineering, University of Connecticut, Storrs, CT 06269, USA; hugo.posada-quintero@uconn.edu

**Keywords:** EEG (electroencephalography), FAA (frontal alpha asymmetry), EDA (electrodermal activity), HRV (heart rate variability), information theory, mutual information, hierarchical clustering, support vector machines, sleep deprivation

## Abstract

Frontal alpha asymmetry (FAA), a pattern of brain activity that reflects the difference in alpha wave power between the left and right frontal areas of the brain, is considered a stable marker for an individual’s tendency to experience either more approach-related or withdrawal-related emotions. On the other hand, electrodermal activity (EDA) measures arousal by tracking changes in skin sweat, which are controlled by the sympathetic nervous system. This study explores the interrelation between EDA features, obtained from time and frequency domains, with FAA by means of the mutual information. Multiple cognitive tasks such as EAT, ship search, PVT and N-Back were analyzed in 10 participants in intervals of two hours over 24 h (12 trials), in which they had to face sleep deprivation conditions. The most informative EDA features about FAA, were used to identify the two main clusters associated to high and low FAA values through the hierarchical agglomerative clustering approach. Once data is labeled, a supervised classifier based on support vector machines (SVMs) is used to identify positive and negative emotional states by using a rigorous one-trial out cross-validation scheme. Results show consistent performance within tasks and trials, achieving accuracy values over 80% on average, giving an important insight about the use of EDA signal as an alternative to the more complex FAA measurement for tracking positive or negative emotional states.

## 1. Introduction

Frontal alpha asymmetry (FAA) has widely been studied as a measurement related to emotional processing, motivation and various psychopathologies. FAA, which is inversely related to cortical network activity, is calculated as the alpha power difference between the right and left hemispheres, particularly in the frontal cortex, allowing the identification of reward-related behaviors when it is high, and avoidance or withdrawal behaviors when it decreases [1,2]. The negative or positive affectivity described by FAA has been associated to mental conditions such as depression, anxiety and stress in many works [3,4,5,6] for analyzing effects in treatments such as behavioral activation [7], for the prediction of treatment effects of major depressive disorders (MDD) in women [8], or in the identification of stressful life events in children with familial risk, suggesting a potential protective role of left frontal activation [9]. In spite of the relevant results shown in many works, some considerations, especially in depression analysis, have been reported in terms of the possible measurement differences induced by EEG recording techniques, age, gender, stress and temperament of individuals, or in EEG sensor locations, i.e., frontal, front-lateral and parietal [10,11,12,13], which suggests a more detailed analysis including EEG not only during rest as traditionally has been performed, but also during emotionally evocative tasks, considering large-scale studies and employing techniques such as multi-modal imaging to link frontal asymmetry to induced emotional states, that is, beliefs about oneself and the world, represented in sadness, happiness, depression, anxiety, and subjective stress, among others [14,15].

On the other hand, electrodermal activity (EDA), a measure of sympathetic nervous system arousal, has been recently studied as a mechanism for the analysis of emotion response [16] and stress detection in normal and sleep deprivation conditions in different cognitive tasks [17], using in most of the cases supervised learning techniques with feature extraction from raw blood pressure, EEG, EDA and ECG data [18,19], and face-emotion identification using deep learning, not only considering the EDA signal but also the phasic (Skin Conductance Response—SCR) and tonic (Skin Conductance Level—SCL) frequency components [20,21]. Since EDA signal extraction and analysis suggests less complexity and costs in comparison with EEG, feature extraction from EDA has gained lots of interest within the research community in recent years for the classification and prediction of stress and depression, which has stimulated the exploration of interrelations between FAA and EDA parameters such as SCR peaks number, SCR amplitude, and SCR rise time, among other SCR event-related features [22].

Beyond these SCR-based features, wavelet-based features have also demonstrated potential for emotions classification due to its ability to capture the non-stationary behavior of EDA signal through its time–frequency analysis. This is exemplified in approaches using the EDA signal for the classification of children and adolescent emotions and social anxiety disorders by means of multiple algorithms such as support vector machines (SVMs) or multi-layer perceptron (MLP), which use in some cases multi-modal data to find relationships between facial expressions and EDA signal changes [23,24,25]. In addition to the standard time–frequency and event-related features, research has also explored the relevance of alternative feature sets for emotion recognition from EDA. This includes statistical features, such as the mean, standard deviation, and Mel-Frequency Cepstral Coefficients (MFCCs) [26]. Furthermore, features adapted from electroencephalography (EEG) analysis, like Hjorth parameters [27] and Higher Order Crossing (HOC) [28], have been translated into the time domain of EDA signals. Among these, statistical features have shown notable promise for discerning emotional states [26].

In spite of the relevant findings described above in terms of feature extraction and emotions classification, it is noticeable that most of the research has focused on the analysis of EEG and EDA signals recorded under resting conditions, without considering their behavior during cognitive tasks and sleep deprivation, two common real-world factors that may predispose individuals to stress, anxiety, and depressive symptoms by overloading regulatory systems and biasing emotional processing toward negative valence. On the other hand, existing studies do not consider the potential relationship between features extracted from the EDA signal and FAA, and thus determine which of these may be more relevant for classifying emotions without the need to capture the EEG signal. In this regard, this work addresses the relationship between multiple EDA features and FAA by analyzing the mutual information between them, across different cognitive activities such as EAT (Error Awareness Task), Ship Search, PVT (Psychomotor Vigilance Task), and N-Back, under sleep deprivation conditions. In contrast to the common statistical tools widely used in the literature to identify signal interactions, such as Pearson’s and Spearman correlation coefficients, ANOVA, and Friedman test, among others, which are limited to the linear scope, by means of the mutual information we perform a more robust analysis that considers both linear and non-linear interactions between EDA and FAA in order to provide new insights about the connection between brain activity and the autonomic sympathetic activity. In addition, we use the most informative EDA features to identify clusters associated to approach-related or withdrawal-related emotions by using hierarchical agglomerative clustering to subsequently create a supervised model that allows to classify both emotional states with high consistency and accuracy within different cognitive tasks requiring high concentration and memory loads, and spatial temporal awareness.

**Figure 1:**
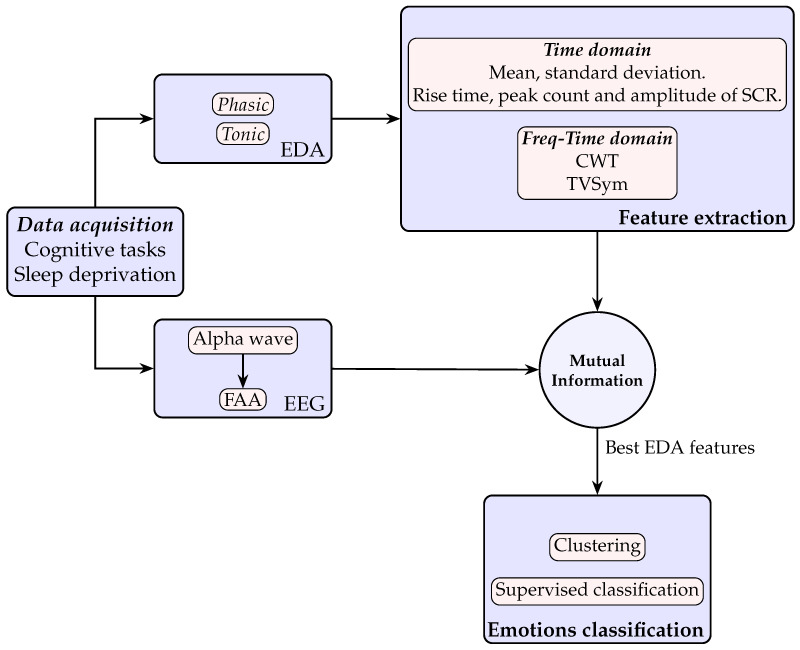
Electrodermal activity (EDA) and electroencephalography (EEG) signals are acquired from participants performing cognitive tasks under conditions of sleep deprivation. The phasic and tonic components of the EDA signal are used to extract time and time–frequency domain features using a moving average window. Mutual information between these EDA features and frontal alpha asymmetry (FAA) is computed to identify the most relevant EDA features. Finally, this refined subset of features is used to classify emotional states by means of clustering and supervised learning methods.

The abovementioned contributions, which, to the best of our knowledge have not been addressed in the literature, provide new insights about the potential of the EDA signal in emotional analysis, similar to the established role of FAA. These contributions, along with the methodology performed in this approach, are summarized in Figure 1. First, EDA and EEG data are obtained under multiple cognitive tasks and sleep deprivation conditions. Second, EDA features are extracted in time and frequency–time domains to analyze their mutual information with FAA and determine which of them have the greatest potential for emotion identification using machine learning elements such as agglomerative clustering and supervised learning. The results demonstrate robust classification performance, with accuracy values over 80%. This confirms the potential of electrodermal activity as a reliable signal for emotion classification.

This paper is divided as follows. Section 2.1, Section 2.2.1, Section 2.2.2, Section 2.2.3, Section 2.2.4, Section 2.2.5, Section 2.2.6 and Section 2.2.7 describe the main concepts related to information theory, performed tasks, EDA features, FAA computation methods, and clustering process used in this approach. Section 2.3 describes the performed methods to calculate the mutual information, the clustering process and the supervised model generation. Finally, in Section 3, Section 4 and Section 6, we present the results and limitations and analyze them.

## 2. Materials and Methods

### 2.1. Data Acquisition Protocol

This research, conducted with the approval of the Institutional Review Board (Protocol # H16-034) of the University of Connecticut, involved ten healthy volunteers (7 male, 3 female) aged 25 to 35 with no reported sleep disorders. The experimental protocol required participants to perform a series of cognitive tasks, detailed in Section 2.2.4, at two-hour intervals over a 25 h period. Data collection commenced at 10:00 a.m., beginning with the EAT task and followed sequentially by ship search, N-Back, and PVT tasks. The dataset comprised EEG signals, analyzed across all five frequency bands, and EDA signals, from which both phasic and tonic components were extracted. Furthermore, ECG signals were recorded throughout each trial to assess the impact of sleep deprivation and cognitive tasks on heart rate variability (HRV), which is used as one of the features to be related with FAA in this study. All participants were instructed to arrive at the laboratory within two hours after waking up and completed a pre-experiment questionnaire to verify their prior night’s sleep quality and duration. ECG data were acquired using an HP 78354A monitor (Hewlett-Packard), whereas EDA signals were captured with an FE116 galvanic skin response amplifier (ADINSTRUMENTS), which was calibrated to zero before each recording session. EEG signals were obtained using an actiCHamp amplifier (Brain Products GmbH, Gilching, Germany) in conjunction with an EasyCap electrode system (EasyCap GmbH, Herrsching-Breitbrunn, Germany).

Prior to the test, participants were fitted with EEG and EDA sensors following a five-minute setup period. The EEG was recorded using a ten-electrode cap, with two reference electrodes on the ears and the remaining eight positioned to capture channels $F_{p2},F_{7},F_{8},O_{1},O_{z},P_{z},O_{2},T_{7}$ and $T_{8}$. The EEG signal was sampled at 200 Hz and bandpass-filtered from 0.5 to 50 Hz. In line with best practices for biosignal acquisition [29], we maintained EEG electrode impedance below 5 kΩ using conductive gel to ensure robust signal quality. On the other hand, EDA was sampled at 400 Hz and measured with stainless steel electrodes on the non-dominant hand’s middle and index fingers, and its tonic and phasic components were separated via the convex optimization method described in [30].

### 2.2. Preliminaries

#### 2.2.1. Mutual Information

**Figure 2:**
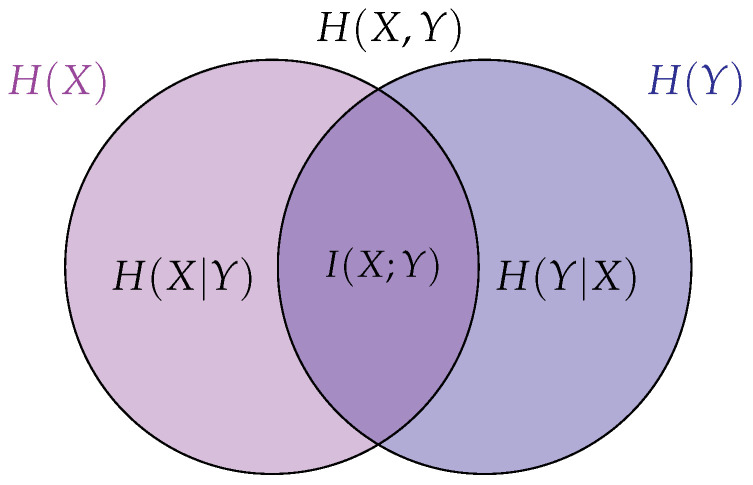
Information theory measures. H(X) represents the entropy of the random variable X, whereas H(Y) the entropy of Y. The joint entropy H(X,Y) is given by the union of both sets, whereas the conditional entropies can be expressed as H(X|Y)=H(X,Y)−H(Y) and H(Y|X)=H(X,Y)−H(X). The mutual information between X and Y is the uncertainty reduction of X or Y when the other is known, that is I(X;Y)=H(X)−H(X|Y)=H(Y)−H(Y|X).

The mutual information is one of the most relevant and widely used concepts from the information theory developed by Claude E. Shannon [31], not only in communications theory but in areas such as data analysis for multiple disciplines (biology, economy, and engineering, among others) [32,33]. It can be described from the entropy concept, which determines the uncertainty level of a random variable *X* from its probability distribution p(x), through the expression $H\left(X\right)=-\sum_{x\in X}p(x)\log p(x)\;\left[bits\right]$, where p(x)=Pr{X=x},x∈X, with X as the alphabet of *X*. In this sense, the joint entropy can be used to describe the uncertainty between two random variables *X* and *Y* by means of their joint probability distribution p(x,y) as follows:

(1) $$\begin{array}{cc} H(X,Y) & =-\sum_{x\in X}\sum_{y\in Y}p(x,y)\log p(x,y)\\ \; & =H(X)+H(Y|X), \end{array}$$

where H(Y|X) represents the conditional entropy, i.e., the uncertainty about *Y*, when *X* is known. The independent, joint and conditional entropies can be visualized in the Venn diagram of Figure 2. The mutual information can be seen as the intersection between H(X) and H(Y), i.e., it describes the uncertainty reduction about a random variable for having information about the other. In addition to the gain of information, mutual information allows the identification of linear and non-linear relationships between random variables [34], which lead us to think that it is possible to find interrelations between EDA and frontal alpha asymmetry (FAA). Furthermore, mutual information has become a highly relevant measure in neuroscience. Unlike other metrics, it is model-free, meaning it does not require assuming a specific structure (such as a linear or non-linear equation) to characterize the relationship between variables [35].

Mathematically, mutual information depends on the joint and marginal probability distributions of both variables and is given by

(2) $$I\left(X;Y\right)=\sum_{x\in X}\sum_{y\in Y}p\left(x,y\right)\log\frac{p(x,y)}{p(x)\;p(y)}=H\left(X\right)-H\left(X|Y\right)=H\left(Y\right)-H\left(Y|X\right).$$

#### 2.2.2. Electrodermal Activity (EDA)

The electrodermal activity (EDA) reflects the changes in skin conductance produced on the sweat glands by the sympathetic nerve activity. This signal is composed by two components known as Skin Conductance Level (SCL; also known as the tonic component) and the Skin Conductance Response (SCR; also know as the phasic component), which are commonly separated by using the convex optimization approach proposed in [30]. The SCR component, which represents the fast changes in conductance levels, is associated to external stimulus such as cognitive tasks, threatening images or loud tones of different frequencies. On the other hand, the SCL component, which represents the long-term EDA variations, is associated to particular individual emotions and thoughts [17].

#### 2.2.3. Electroencephalography (EEG)

Electroencephalography (EEG) captures the electrical brain activity generated by the synchronized activity of neurons. This signal is composed by delta (1–4 Hz), theta (4–8 Hz), alpha (8–13 Hz), beta (13–30 Hz) and gamma (>30 Hz) waves [17]. In this study, the alpha component has a relevant role since its power allows to determine the frontal alpha asymmetry, an EEG metric used in emotional analysis.

##### Frontal Alpha Asymmetry-FAA

Frontal alpha asymmetry is a physiological metric to measure the activity difference between the right and left frontal areas of the brain, through the power of the alpha component (8–13 Hz) of the EEG signal. This power value is inversely related to cortical activity, which means that when FAA increases, left cortical activation is greater than the right, indicating enhanced approach-oriented or reward-seeking behaviors, and positive affect, rather than avoidance, withdrawal or negative emotions, linked to relative right-frontal activation or lower FAA values [36]. FAA is commonly calculated as the difference between the alpha power of the mid-frontal ($F_{4},F_{3}$), pre-frontal ($FP_{2},FP_{1}$) or the lateral-frontal ($F_{8},F_{7}$) EEG channels [37], by means of the normalized ratio expression $FAA=\frac{R_{power}-L_{power}}{R_{power}+L_{power}}$, or by means of the logarithmic expression $FAA=\log\left(R_{power}\right)-\log\left(L_{power}\right)$, where $R_{power}$ and $L_{power}$ represent the power of the right and left channels, respectively [38], which, in our case, were calculated in the time domain using the expression $\frac{1}{N}\sum_{n=1}^{N}{\left(\alpha_{left}\left[n\right]\right)}^{2}\left[\mu V^{2}\right]$, where $\alpha_{left}\left[n\right]$ represents the band-pass filtered (8-13 Hz) of EEG signal ($F_{7}$ channel) at sample n, and N is the number of samples of each window. The same calculation was performed for the right frontal channel ($F_{8}$) to obtain $R_{power}$. In this approach, we explore the mutual information described in Section 2.2.1 as a FAA metric. We compare it with traditional calculation methods in the multiple cognitive tasks and under sleep deprivation conditions in Section 3.

#### 2.2.4. Performed Tasks

##### Error Awareness Task (EAT)

In our study, this cognitive task takes 5 min during which, a sequence of color names with colored letters is presented. Each image is visible during 900 ms, and the interval between them is 600 ms. A ’Go’ trial event occurs when the participant press a button indicating that the color of the letters and the color name match (e.g., the word ’Red’ appears in red color). On the contrary, a ’No Go’ trial occurs when the participant avoids to press the button due to the non-correspondence between the color name and the color of the letters, or when the same color name appears in two consecutive trials [39,40]. The ’No Go’ trials allow the participants to behave more attentively than repetitively, submitting them to stressful situations, increasing their alertness condition [41].

##### Ship Search

This 20 min task is designed to assess the spatial and temporal awareness of individuals. Participants are required to monitor an interactive screen simulating a periscope view over the ocean and identify the appearance of a ship. They must press the space bar at the moment of appearance and verbally report its coordinates [19].

##### N-Back

This 10 min memory task requires participants to identify whether an audible tone matches one presented n-steps earlier in a sequence. Tones of varying frequency and duration are commonly played at 3 s intervals through two speakers positioned in front of the participant. Using designated computer keys, participants must indicate whether each tone is similar to the n-th previous one. The value of n is adaptively adjusted based on individual performance, increasing or decreasing the task difficulty. To further elevate complexity, the audible stimuli may be paired with simultaneous visual cues [19]. Due to these cognitive demands, this protocol is regarded as a high-load working memory task [42].

##### Psychomotor Vigilance Task (PVT)

This task takes 10 min and requires the participants to click the left mouse button as quickly as possible whenever a number appears on the screen at random intervals between 2 and 10 s. All participants performed the Psychomotor Vigilance Task (PVT) on the same computer using freely available software as described in [43,44]. The PVT is widely used to assess attentional degradation under sleep deprivation conditions by measuring reaction time (RT) to repetitive visual stimuli.

#### 2.2.5. Hierarchical Agglomerative Clustering

This type of clustering requires a pair-wise distance matrix mxm, calculated between each pair of training points and considering each feature or dimension. For the squared Euclidean distance case, this is calculated using the expression $d{\left(x,y\right)}^{2}=\sum_{j=1}^{m}{\left(x_{j}-y_{j}\right)}^{2}$, where *j* refers to the jth feature, and x,y to a pair of training points of the *m*-dimensional space. Agglomerative clustering initializes each training sample as its own cluster, resulting in an initial state of *m* clusters. Then, the closest clusters, which can be determined depending on the distance between their most closest members (single linkage), their furthest members (complete linkage) or the average distance between all their member pairs (average linkage) are merged. The distance matrix is iteratively updated until no more clusters are available for merging [45]. This method offers several advantages, such as the generation of hierarchical dendrograms to visualize data structure based on cluster distances, which can be used to determine the number of clusters for a specific dataset. Furthermore, it does not require pre-specifying the number of clusters, a key limitation of centroid-based algorithms like K-Means. In our approach, this clustering technique allows the identification of relationships between EDA features and FAA. We present and discuss these results in Section 3.

#### 2.2.6. EDA Features

##### Time Domain Features

In EDA signal analysis, multiple event-related features have been used for emotion analysis. These features are typically extracted from time windows where the SCR value is conditioned to a threshold amplitude, commonly set at 5 μS, in order to discard small and non-representative levels [46]. Between the most used SCR-based features, we have the SCR rise time (the interval between the SCR onset and its peak), the SCR peak count (the number of SCR peaks in a window), and the SCR amplitude (the magnitude of the response, which is associated with the intensity of sympathetic arousal). From a statistical perspective, the mean and standard deviation both of the EDA signal and its tonic and phasic components have been explored [26,47].

##### Time–Frequency Domain Features

Given the non-stationary behavior of the EDA signal, we employ time–frequency analysis tools. Specifically, our approach utilizes wavelet analysis and the highly sensitive index of sympathetic tone proposed in [48].

##### Continuous Wavelet Transform

Continuous wavelet transform (CWT) provides a detailed analysis of frequency behavior in time, specifically by means of a scaleogram. In contrast to the discrete wavelet transform, especially used for de-noising and frequency component extraction [26], CWT provides more details for feature extraction, especially when complex components are used as in the complex Morlet (C-Morlet) case [23], which we use in this approach. This transform employs a zero-mean, finite-energy mother wavelet constructed as the product of a Gaussian envelope and a complex carrier signal. Its expression is given by

(3) $$\psi^{*}\left(\eta \right)=\frac{\exp\left(\frac{-\eta^{2}}{f_{b}}\right)}{\sqrt{\pi f_{b}}}\exp\left(j2\pi f_{c}\eta \right),$$

where $f_{b}$ is the non-dimensional bandwidth, $f_{c}$ is the non-dimensional center frequency and η is a non-dimensional time parameter [49,50]. The term $\frac{1}{\sqrt{\pi f_{b}}}$ is used to ensure the finite energy condition of $\psi^{*}\left(t\right)$, since, $\frac{1}{\sqrt{\pi f_{b}}}\int_{-\infty }^{\infty }{\left|\exp\left(\frac{-t^{2}}{f_{b}}\right)\right|}^{2}{\left|\exp(j2\pi f_{c}t)\right|}^{2}dt=\frac{1}{\sqrt{\pi f_{b}}}\sqrt{\frac{\pi f_{b}}{2}}=\frac{1}{\sqrt{2}}$. Additionally, this term acts as a normalization factor that stabilizes wavelet energy across different $f_{b}$ values. It prevents wavelet coefficients from being artificially amplified or attenuated due to changes in the wavelet energy, ensuring that their magnitudes reflect genuine signal properties. $\psi^{*}\left(t\right)$ is scaled by $a\in R^{+}$, and translated by b∈R along the time axis, to generate a set of daughter wavelets that are matched with the original signal x(t) by means of the expression

(4) $$\mathrm{CWT}\left(a,b\right)=\int_{-\infty }^{\infty }x\left(t\right)\cdot \frac{1}{\sqrt{a}}\psi^{*}\frac{\left(t-b\right)}{a}\;dt,$$

where the term $\frac{1}{\sqrt{a}}$ ensures that every daughter wavelet has the same energy as the mother wavelet. The wavelet is stretched when a≥1, making it correlate with lower-frequencies (broader features), and it is squeezed when a≤1, making it correlate with higher-frequencies (sharper features).

##### Time-Varying Spectral Amplitudes- TVSymp

TVSymp is a highly sensitive index of sympathetic tone obtained by using the variable frequency complex demodulation technique (VFCDM) [51], which uses a bank of low-pass filters to decompose the EDA signal in a suite of band-limited signals that, by means of the Hilbert transform, provide instantaneous frequency, amplitude and phase values within each frequency band [48]. To understand this, let us consider a narrow-band signal $x\left(t\right)=dc\left(t\right)+A\left(t\right)\cos[2\pi f_{o}t+\phi \left(t\right)]$ with center frequency $f_{o}$, instantaneous phase ϕ(t), instantaneous amplitude A(t), and direct current component dc(t). The frequency-shifting property (demodulation property [52]) can be applied to x(t) to obtain

(5) $$z\left(t\right)=dc\left(t\right)e^{-j2\pi f_{o}t}+\frac{A(t)}{2}e^{j\phi (t)}+\frac{A(t)}{2}e^{-j[4\pi f_{o}t+\phi \left(t\right)]}.$$

Applying an ideal low-pass filter (cutoff $f_{c}<f_{o}$) to z(t) results in $z_{LP}\left(t\right)=\frac{A(t)}{2}e^{j\phi (t)}$, from which we can recover the instantaneous amplitude

(6) $$A\left(t\right)=2|z_{LP}\left(t\right)|,$$

and the instantaneous phase

(7) $$\phi \left(t\right)=\arctan\left(\frac{Im\{z_{LP}\left(t\right)\}}{Re\{z_{LP}\left(t\right)\}}\right).$$

Now, considering the case of a time-varying frequency, we have the following expressions:

(8) $$x\left(t\right)=dc\left(t\right)+A\left(t\right)\cos\left(\int_{0}^{t}2\pi f(\tau )d\tau +\phi \left(t\right)\right),$$

and

(9) $$z\left(t\right)=dc\left(t\right)e^{-j\int_{0}^{t}2\pi f(\tau )d\tau }+\frac{A(t)}{2}e^{j\phi (t)}+\frac{A(t)}{2}e^{-j[\int_{0}^{t}4\pi f(\tau )d\tau +\phi \left(t\right)]}.$$

Again, if z(t) is filtered with an ideal low-pass filter with $f_{c}<f_{o}$, the instantaneous amplitude and phase can be obtained using Equations (6) and (7).

Given the mathematical framework established above, the implementation of VFCDM consists of the following main steps:

1. A set of center frequencies is given by $f_{oi}=\left(i-1\right)\left(2F_{\omega }\right),\;i=1,2,3,\ldots ,int\left(\frac{f_{max}}{2F_{\omega }}\right)$, where $F_{\omega }$ is the bandwidth of a LPF (FIR) with length $N_{\omega }$. The bandwidth between neighboring center frequencies is $2F_{\omega }$ and $f_{max}$ is the highest signal frequency.
2. Using Equations (6) and (7), extract the most representative frequency within the bandwidth, and repeat the process over the entire frequency band by incrementing $f_{oi}$.
3. Decompose the signal into sinusoidal modulation components using the variable frequency approach, which leads to the expression
  (10) $$x\left(t\right)=\sum_{i}d_{i}=dc\left(t\right)+A_{i}\left(t\right)+\cos\left[\int_{0}^{t}2\pi f_{i}\left(\tau \right)+\phi \left(t\right)\right].$$
4. For each sinusoidal modulation component, calculate the instantaneous frequency $f\left(t\right)=\frac{1}{2\pi }\frac{d\phi (t)}{dt}$ and the instantaneous amplitude $A\left(t\right)=\sqrt{X^{2}\left(t\right)+Y^{2}\left(t\right)}$, using the Hilbert transform $Y\left(t\right)=\frac{1}{\pi }\int \frac{X(\tau )}{t-\tau }d\tau$.
5. Finally, obtain the time–frequency representation of the signal using the estimated instantaneous frequencies and amplitudes.

By computing the Hilbert transform of Equation (10) at every time point for each of the low-pass filtered frequency components, as detailed in Equations (6) and (7), we obtain the complete time–frequency spectrum. Consequently, VFCDM yields a high-resolution time–frequency spectrum along with precise amplitude information. The TVSymp index, derived from VFCDM applied to EDA signals, exhibits high values in the 0.08–0.24 Hz band, reflecting sympathetic nervous system activation by stressors [48]. This work examines the relationship between the TVSymp index and FAA using mutual information, evaluating this association across a range of cognitive tasks and under the physiological challenge of sleep deprivation.

#### 2.2.7. Heart Rate Variability (HRV)

The HRV is an electrocardiogram (ECG) signal metric, used to determine the variation between heartbeats. This variability is a normal function of the autonomic nervous system (ANS), which balances the “fight-or-flight” (sympathetic) and “rest-and-digest” (parasympathetic) responses. A higher HRV generally indicates better health, fitness, and stress resilience, whereas a lower HRV can be a sign of stress or illness [53,54]. The HRV calculation is performed by multiple methods, which range from time-domain (SDNN, RMSSD, pNN50, among others), frequency domain (VLF, HF, LF, among others). In this study, we use the Root Mean Square of Successive Differences (RMSSD) measure, calculated as $\mathrm{RMSSD}=\sqrt{\frac{1}{N-1}\sum_{i=1}^{N-1}{\left(RR_{i+1}-RR_{i}\right)}^{2}}$, where $RR_{i}$ is the duration of the *i*-th R-R interval (in milliseconds) and *N* is the total number of successive R-R intervals, with R being the amplitude peak of the QRS ECG signal interval. Compared to other measures, RMSSD is a more specific indicator of parasympathetic nervous system activity. It is therefore well-suited to estimate the idle mediated fluctuations in heart rate [55]. It provides statistical robustness, which improves the HRV analysis in short-term time windows as our study requires [56]. In this work, we use HRV as an additional parameter to identify EDA features related to FAA by means of the mutual information.

### 2.3. Mutual Information Between FAA and EDA-ECG Features

#### 2.3.1. Feature Extraction

**Figure 3:**
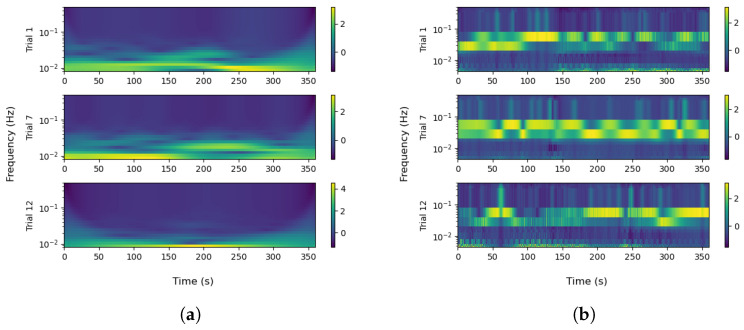
Scaleograms for EDA components obtained from the continuous wavelet transform (in order to show significant changes, only trials 1, 7 and 12 are considered). Resulting wavelet intensity, presented in the right color-bars, is consistently high in Trial 7, i.e., for all the trial duration (around 10 PM), when participants experienced greater cognitive fatigue and stress from sleep deprivation, compared to Trials 1 and 12. Frequency is presented in logarithmic scale as suggested in [49]. (**a**) Tonic component; (**b**) phasic component.

For the feature extraction process, we use the moving average window method [57,58], with a window duration of 64 s (13,000 samples for a sampling frequency of 200 Hz) as required to extract the TVSymp index [48]. On each iteration, each window includes 325 (≈1.62 s) new samples and discards 325 old samples of the data for statistical calculations. The computed statistical values involve the mean and standard deviation for the tonic and phasic components of the EDA raw data, considering each cognitive task and trial, and the data of all the 11 participants. The mean and standard deviation are also calculated in the same time window for the SCR-based features (event-related), and for the frequency–time domain features described in Section 2.2.6. In the continuous wavelet case, we use a C-Morlet wavelet with $f_{b}=1.5$ Hz and $f_{c}=1$ Hz and 25 different scale values from 800 to 10,000 corresponding to a frequency band from 0.005 to 0.5 Hz in order to cover the spectrum of tonic and phasic components (<0.05 Hz for tonic and 0.05–0.15 Hz for phasic). The translation time is of 1 s. In this sense, we have 25 different frequency values for each second of the 4 analyzed cognitive tasks and their corresponding 12 trials. The results for trials 1, 7 and 12 for EAT task are shown in the scaleogram of Figure 3. The CWT coefficients obtained for each second are finally used to calculate the mean and standard deviation of the wavelet transform, both for phasic and tonic components. Notice how the magnitude of the frequency components evolves over the course of each trial, with more sustained activity observed in trial 7 for both the tonic and phasic EDA components, when participant tiredness peaked due to sleep deprivation. This finding highlights the value of wavelet-based features in detecting stressful situations or negative emotions associated with fatigue.

On the other hand, FAA and HRV are also obtained for each time window. In the FAA case, the normalized ratio, logarithmic and mutual information approaches are used (see Section Frontal Alpha Asymmetry-FAA). The HRV is calculated as described in Section 2.2.7.

#### 2.3.2. Mutual Information

**Figure 4:**
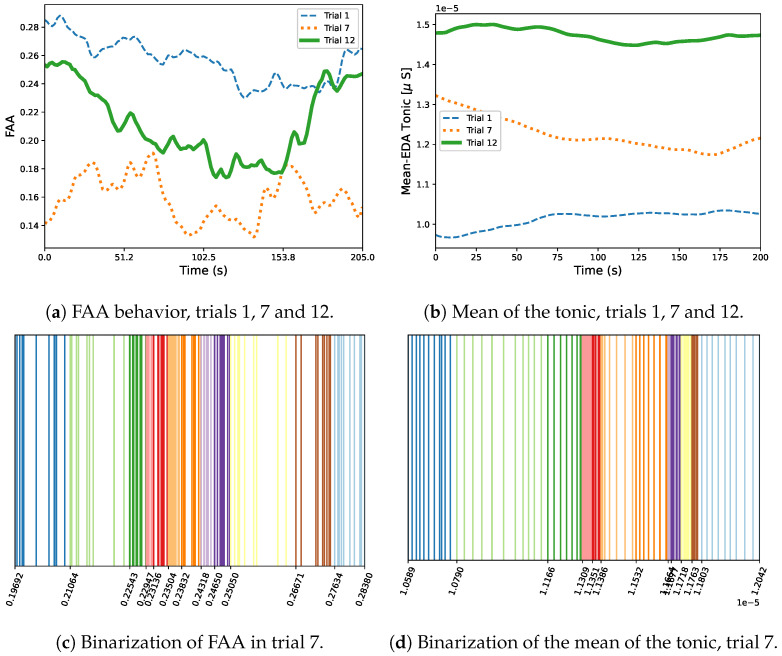
Binarization process for FAA and the mean of the tonic. (**a**) FAA data obtained using the average window method in trials 1, 7 and 12. Observe how FAA decreases significantly at 10 pm (trial 7), when sleep deprivation increases fatigue and stress. (**b**) Mean of the tonic component of EDA for trials 1, 7 and 12. (**c**) Binarization for FAA (trial 7). (**d**) Binarization of the mean of tonic data (trial 7). Some trials are narrower than others in both signals.

According to Equation (2), mutual information requires marginal and joint probability values for the evaluated random variables, which, in our case represent the set of feature values obtained over the time windows. This probability calculation is performed through the uniform count binning process presented in a previous work [17], where 12 uniform count bins or states are generated to contain different continuous values. The first step consists of maximizing the entropy value and consequently the mutual information by defining each state probability with the expression $p\left(s\right)=\frac{N(s)}{N_{obs}}$, where N(s) and $N_{obs}$ are the number of observations in a specific bin, and the total number of observations, respectively. Thus for example, in the FAA and mean tonic values obtained through all the windows (Figure 4a,b, respectively), we have 127 samples distributed in 12 bins, each one with ≈10 samples. In this sense, $p\left(s\right)=\frac{10}{127}\approx 0.083$, i.e., the probability distribution for the 12 bins is uniform, which maximizes the entropy. This uniform distribution makes some bins narrower than others, which is shown in Figure 4c,d, for FAA and tonic mean, respectively. The joint probability distribution is calculated considering the proportion of data points that fall into a specific combination of bins.

**Figure 5:**
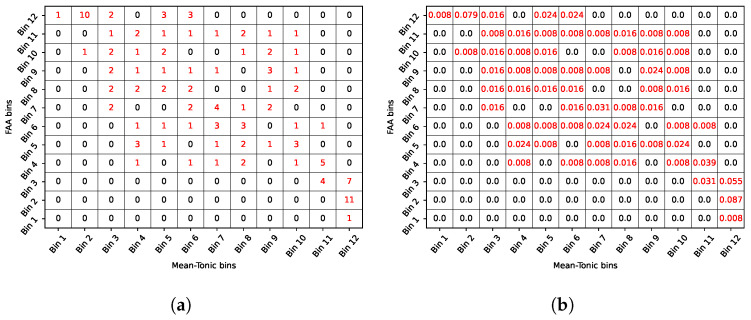
Bin combination for FAA and mean tonic component. (**a**) Joint frequency; (**b**) joint probability.

Figure 5a,b show, respectively, the joint frequency and the joint probability for the FAA and mean tonic data in a given window. Once the joint probabilities are calculated, the mutual information can be obtained for every pair of features. For the particular case of FAA and mean tonic, the mutual information is 0.9818 [bits]. To address the potential bias, inherent to discrete information estimates and finite samples, we apply the Miller–Madow bias correction [59,60]. This correction is well-established for entropy and mutual information estimation and accounts for the fact that, with limited data, random coincidences can create spurious correlations that distort mutual information values.

## 3. Results

**Figure 6:**
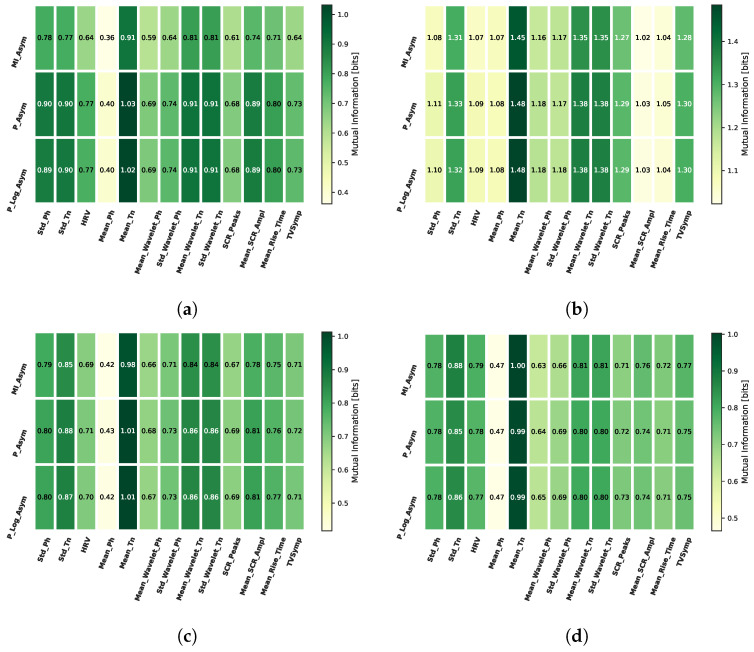
Average over trials and individuals of mutual information between features and FAA. The three FAA approaches are considered, showing similar results for each case. (**a**) EAT; (**b**) N-Back; (**c**) ship search; (**d**) PVT.

In Figure 6, we show the mutual information values found between the three types of alpha asymmetry described in Section Frontal Alpha Asymmetry-FAA and the extracted features, for all the analyzed cognitive tasks, averaged over all trials and individuals. Here, we can observe that the values are similar for the three cases, which demonstrates the consistency between the asymmetry types. In general, the mean of the tonic component has the highest values of mutual information with FAA, especially for the N-Back task.

**Figure 7:**
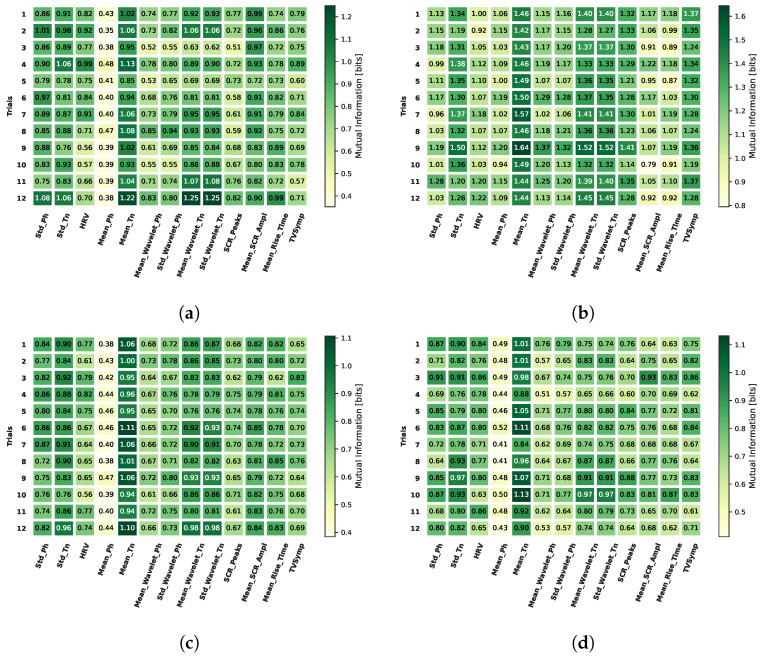
Average over individuals of mutual information between the analyzed features and normalized ratio FAA for the 12 trials and all tasks. (**a**) EAT; (**b**) N-Back; (**c**) Ship search; (**d**) PVT.

Additionally, in Figure 7, we show the mutual information between FAA and all the studied features for all trials and all tasks, averaged over all the individuals. Again, it is noticeable that the features having the highest mutual information with FAA are the mean and standard deviation of the tonic component, along with the mean and standard deviation of wavelet coefficients and the TVSymp index. In this sense, from the 13 evaluated features, we select as the main features for our analysis the six with the highest mutual information values, which are the mean and standard deviation of the tonic component (Mean_Tn and Std_Tn), the mean of the wavelet coefficients for the tonic component (Mean_WL_Tn), the SCR amplitude (SCR_Ampl), the SCR rise time (SCR_RiseT), and the TVSymp index.

To assess the statistical significance of the relationships between FAA and EDA features, we employed two complementary approaches. First, we conducted a permutation-based analysis using mutual information as the test statistic, with 10,000 surrogate permutations. These permutations were performed, individually, for each of the six selected EDA features, and considering trials 1, 7 and 10 for independent participants that were randomly chosen. This analysis revealed highly significant dependencies between FAA and the six EDA features (all p<0.0002), indicating robust non-linear relationships.

Second, to examine linear associations specifically, we computed Pearson’s correlation coefficients between FAA and the same EDA features for randomly selected individuals in Trials 1, 7 and 10. As shown in Table 1, the strength and direction of these linear relationships varied substantially across individuals and features. While some correlations were negligible (e.g., Participant 1, Trial 7: r=0.0064, and p=0.936), most of them reached strong statistical significance. These results demonstrate that EDA features can effectively guide the clustering process to distinguish between emotional states.

**Figure 8:**
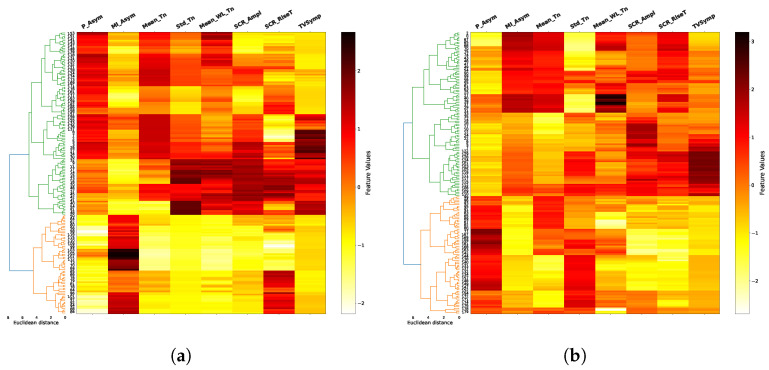
EAT dendograms. For this memory-demanding cognitive task, positive and negative values of FAA (P_Asym) are associated, respectively, to positive and negative values of the mean, standard deviation and wavelet magnitude of tonic component (Mean_Tn, Std_Tn, Mean_WL_Tn). This association allows to identify the two clusters shown in green and orange in the left side using only EDA features. FAA values are shown just for illustrative purposes. (**a**) Trial 1; (**b**) Trial 8.

**Figure 9:**
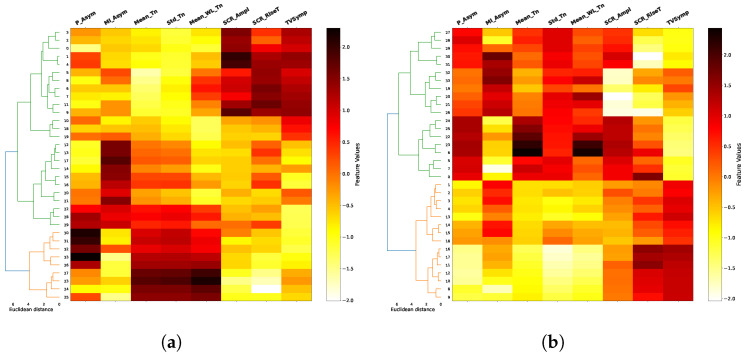
N-Back dendograms. For this alertness task, the relationship between FAA (P_Asym) and the mean, standard deviation and wavelet magnitude of tonic component (Mean_Tn, Std_Tn, Mean_WL_Tn) is also visible, especially in high trials. In contrast to the EAT case, the feature TVSymp exhibits a remarkable relationship with the FAA. (**a**) Trial 8; (**b**) Trial 12.

**Figure 10:**
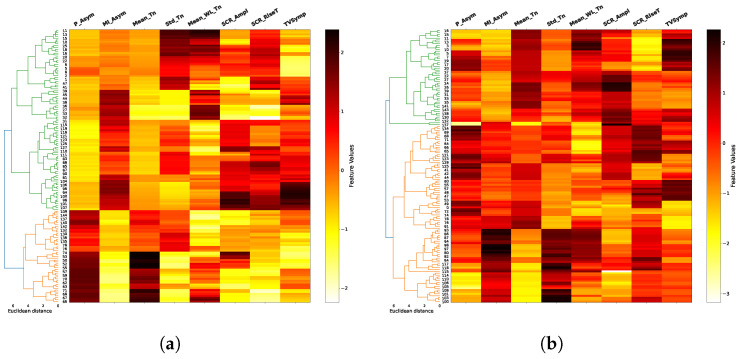
Ship search dendograms. For this spatial–temporal task, the relationship between FAA and the main EDA features is maintained, especially in the initial trials. As in all cognitive tasks, FAA (P_Asym) is not used in the clustering process. (**a**) Trial 8; (**b**) Trial 12.

**Figure 11:**
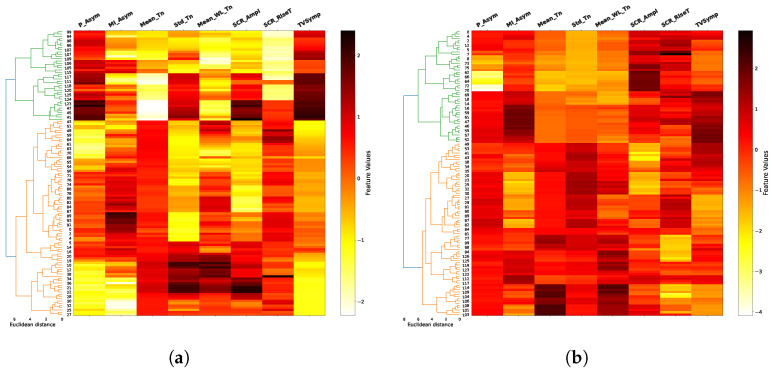
PVT dendograms. For this vigilance awareness task, the relationship between FAA and EDA features is similar to the one exhibited in the ship search case, i.e., it is more distinguishable in initial trials. As in the previous cases, clustering is performed only using EDA features. FAA values are included only for illustrative purposes. (**a**) Trial 1; (**b**) Trial 12.

The results of the agglomerative clustering process are shown in dendograms of Figure 8, Figure 9, Figure 10 and Figure 11, for each task and for combinations of two trials between 1, 8 and 12 (in addition to the space limitations, we selected this set of trials considering the difference of fatigue and stress between them experienced by the individuals during the test). It is noticeable how the two main clusters (green and orange), obtained after a previous data standardization, are primarily separated by the mean of the tonic component (Mean_Tn), SCR amplitude (SCR_Ampl) and TVSymp index, especially for EAT. In the majority of cases, the Euclidean distance between clusters exceeds six, indicating a well-defined and consistent cluster structure. This clear separation, combined with the multiple-trial and multiple-task structure of our experimental design, supports the stability and reproducibility of the clustering solution. The FAA data, both the normalized ratio (P_Asym) and the mutual information (MI_Asym) approaches, are included in dendograms only for illustrative purposes since they were not included in the clusterization algorithm. In general, clusters begin to be less clear in higher trials as in the case of trial 12 in PVT and ship search tasks.

The data labeling produced by agglomerative clusterization allows the use of supervised learning algorithms for prediction. In our case, we use support vector machines due to the good results presented in previous works in physiological signal analysis [23,61]. In this sense, we built a dataset for each cognitive task having as features the ones with higher mutual information with FAA (Mean_Tn, Std_Tn, Mean_WL_Tn, SCR_Ampl, SCR_RiseT, TVSym index). To ensure robust evaluation, we implemented a leave-one-trial-out cross-validation prediction strategy. This approach uses 11 trials for training and reserves one trial for testing, cycling through all 12 trials. The complete set of results from this procedure is described in Table 2, which shows the percentage of coincidence between Class 1 and Class 2 labels with positive and negative values of P_FAA (normalized ratio FAA), respectively.

**Figure 12:**
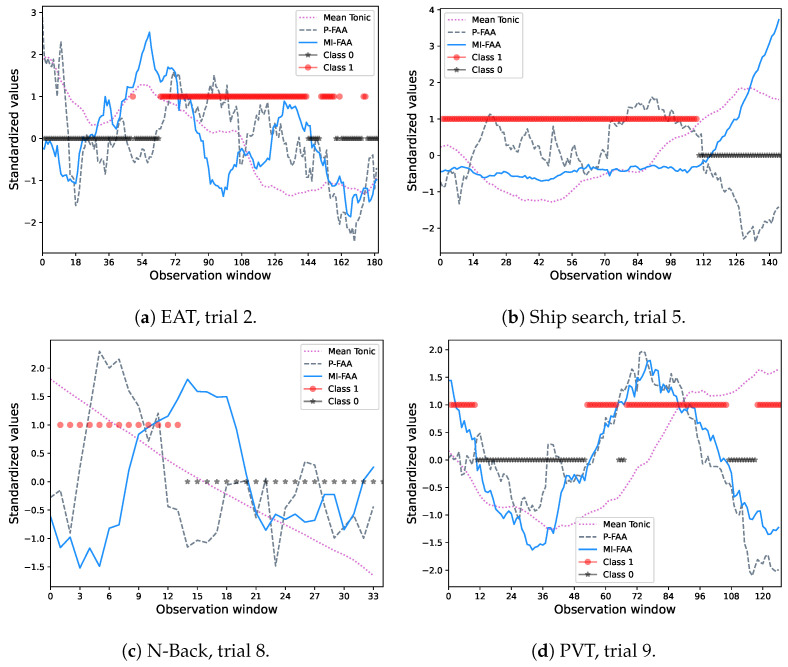
Relationship between FAA (P_FAA and MI_FAA) and the mean of the tonic component across multiple observation windows tasks and trials. Trials are divided in multiple observation windows, each 65 s as we described in Section 2.3, in which feature values were extracted. Red dots represent positive FAA values (reward-related behavior), whereas black stars the negatives (avoidance or withdrawal behavior). Observe how, most of the time, P_FAA positive and negative values correspond to classes 1 and 0, respectively. (**a**) Case of trial 2 of the EAT task. (**b**) Case of trial 5 of the ship search task. (**c**) Case of trial 8 of N-back task. (**d**) Case of trial 9 of PVT task.

On the other hand, Figure 12 shows a comparison between the prediction process (Class 1 in red, Class 2 in black) and some relevant signals such as the P_FAA and MI_FAA (normalized ratio and mutual information approaches, respectively) for some validation trials. Observe how Class 1 is predominantly associated with positive P_FAA values, whereas Class 2 is primarily associated with negative values across most of the observation windows. In general, average percentage results exhibit better performance for EAT and N-Back tasks (≈90%) than ship search and pvt (≈80%) (Table 2). These results suggest better classification accuracy in tasks demanding high memory and alertness conditions than in tasks related to spatial temporal and vigilance awareness.

## 4. Discussion

The results shown in Figure 6 and Figure 7 illustrate the mutual information between multiple EDA features and HRV with FAA. Among these features, the mean of the tonic component of EDA (Mean_Tn), demonstrated to be the most informative about FAA, which provides a relevant insight about an alternative form to study physiological states commonly analyzed with FAA. Additionally, other tonic-based features such as the mean and standard deviation exhibit high mutual information values both in time and time–frequency domains. This increase suggests that the overall level of sympathetic arousal, reflected in tonic activity, is more closely associated with frontal alpha asymmetry (FAA) than rapid, event-related phasic responses. Regarding the TVSymp index and the SCR rise time (SCR_RiseT), these EDA features also demonstrate substantial mutual information with FAA, particularly during the N-Back task, which is a highly memory-demanding task.

On the other hand, the two main clusters (the two class labels) shown in dendograms in Figure 8, Figure 9, Figure 10 and Figure 11 have a clear delimitation given by the positive and negative values of FAA in most of the cases. Additionally, there is a great influence from EDA features such as the mean and standard deviation of tonic component (Mean_Tn, Std_Tn), the mean of wavelet coefficients of tonic (Mean_WL_Tn) and TVsymp index. The observed strong relationship between the tonic component of EDA and FAA aligns with the results reported in [17], which also associate the skin conductance level (SCL) with specific individual emotions and thoughts, a psychological domain to which FAA has been connected. Regarding the TVSymp feature, obtained through the high-resolution time–frequency spectrum technique VFCDM, it exhibits high values in the 0.08–0.24 Hz band [48], a range more closely aligned with the phasic component of EDA. This suggests that certain behaviors not captured by the proposed phasic features may be extracted from this parameter, particularly when analyzing direct sympathetic responses to stimuli.

Unlike conventional event-related EDA features, such as the mean and standard deviation of the tonic component or the SCR rise time, wavelet-based features offer a simultaneous time–frequency representation of the EDA signal. Specifically, they provide information about power density across the analyzed frequency spectrum (0.005–0.5 Hz) and how this spectral composition evolves over time. This allows the identification of which frequency bands are most active under different experimental conditions, such as during states of fatigue or cognitive load (Figure 3). In the clustering process, the mean of the wavelet transform for the tonic component (Mean_WL_Tn) played a notable role in cluster separation in the majority of cases (Figure 8, Figure 9, Figure 10 and Figure 11). Although this feature reflects spectral intensity over time rather than conventional event-related parameters (e.g., signal amplitude, rise time, and peak count, among others), it consistently exhibited similar behavior to these features in relation to FAA throughout the results. This consistency further supports the robustness of wavelet-based features as complementary indicators of the relationship between EDA and FAA.

In terms of cognitive tasks and considering FAA values, clusters are more distinguishable for tasks associated to high memory demands and alertness conditions, such as EAT and N-Back. In contrast, they are less differentiable, specifically in high trials, in tasks like PVT and ship search, which are related to spatial temporal and vigilance awareness. As previously mentioned, FAA measures are included in the dendrograms for illustrative purposes, helping to identify their relationship with EDA features. In this sense, the dendrograms reveal that the normalized FAA ratio (P_Asym) or the mutual information based FAA (MI_FAA) and the mean tonic component (Mean_Tn) can exhibit two distinct patterns within a cluster, i.e., they can both show high values, or one can be high while the other is low (e.g., trials 1 and 12 of EAT). Despite this variability, the cluster division is consistent with what would be achieved using P_Asym since the mutual information is a measure with the ability to consider these non-linear relationships.

In relation to the mutual information-based FAA (MI_FAA), meaningful behavior is discernible in terms of clusters definition since it exhibits both positive and negative relationships with P_Asym and Mean_Tn, especially in EAT and ship search tasks, which provides insights about the potential of this measure in terms of emotions classification. On the other hand, due to this non-linear relationship, it is not possible to distinguish positive and negative emotions from the MI_FAA sign as we do with P_Asym (Figure 12). However, considering the nature of mutual information, low values of MI_FAA suggest high FAA asymmetry, which is visible for ship search and N-Back tasks (Figure 12b,c, respectively). This consistent behavior in specific contexts proposes further research in order to clarify its generalization.

As we mentioned before, the clustering procedure, based only in EDA features, revealed two primary classes corresponding to high and low FAA values. These class labels were subsequently used to train a support vector machine (SVM) model for predicting FAA levels using only EDA features. In this regard, the results shown in Figure 12 and Table 2 demonstrate a good performance in terms of accuracy, in spite of the notable non-linear variation between FAA and features such as the mean of the tonic component (Mean_Tn). First, Figure 12a–d show the classification process through the observation windows (variation in time), where Class 1 (Red dots) is associated to positive FAA values (normalized FAA ratio-P_Asym, dashed lines colored in gray), whereas Class 2 (Black stars) is associated to negative FAA values. Additionally, MI_FAA behavior and Mean_Tn are depicted in solid blue and dotted cyan, respectively. Notably, these variables do not follow a linear relationship with P_Asym, as we have described previously. Second, Table 2 shows the model consistency in terms of accuracy across all trials in the leave-one-trial-out cross-validation strategy.

In terms of statistical significance, the analysis of both non-linear and linear relationships between EDA features and FAA, assessed via mutual information and Pearson’s correlation, respectively, revealed highly significant dependencies, with particularly strong robustness observed in the non-linear case (p<0.0002). In contrast, linear correlations exhibited some variability in both strength and direction across individuals and features (Table 1). While most of these correlations reached statistical significance, some were negligible (e.g., Participant 1, Trial 7: r=0.0064, p=0.936). These findings underscore the value of mutual information as an appropriate tool for detecting relevant relationships that may be overlooked by conventional correlation measures. The use of a permutation surrogation approach for each of the six most representative EDA features, supports the statistical validity of our analysis, as each feature generated its own empirical null distribution through 10,000 permutations. The results showed that all six EDA features reached statistical significance with p<0.0002, providing strong evidence against the null hypothesis. Notably, the probability of observing six independent features all yielding p<0.0002 by chance is very low. As a further conservative test, we applied the Bonferroni correction for multiple comparisons (corrected $\alpha =\frac{0.05}{6}=0.0083$). Even under this most rigorous criterion, all *p*-values remain below the corrected threshold, confirming that our findings are robust to multiple comparisons correction.

In general terms, the abovementioned results have demonstrated robust performance across all cognitive tasks and levels of sleep deprivation, indicating that both cognitive load and fatigue contribute to negative emotional states. This effect was most pronounced during late-night hours when fatigue reached its highest levels (this effect is also shown in Figure 4a).

## 5. Limitations and Future Directions

As we have mentioned, the mutual information-based FAA (MI_FAA) exhibits potential for emotions classification due to its non-linear relationship with the normalized FAA ratio (P_Asym) and the mean tonic component (Mean_Tn) depicted in dendograms in Figure 8 and Figure 9. However, as is noticeable in Figure 12, it is not completely generalizable that low MI_FAA values (negative values when they are standardized) are associated to high asymmetry as expected, considering the mutual information nature. In this sense, further work is necessary to obtain concluding evidence in this regard. To extend the detectable spectrum of emotions, future work could integrate visual, audible, and cognitive stimuli, using methods like facial expression detection while simultaneously recording EDA and FAA signals. This multi-modal approach would allow emotional states to be classified according to established psychological frameworks, such as the circumplex model of affect proposed in [62].

Furthermore, our findings can be integrated with recent advances involving IoT, artificial intelligence, and micro-electro-mechanical system (MEMS) sensors for real-time physiological signal acquisition [63]. Such integration holds particular promise for remote health monitoring applications, especially in elderly populations. Future studies could also investigate the specific contribution of time and time–frequency features by comparing model performance with and without them, thereby providing a more granular understanding of their role in emotion recognition based on the EDA signal.

From the perspective of EDA features, the frequency range of TVSymp suggests a relationship with the phasic component that was not evident in the clustering results. While the phasic signal itself was not strongly associated with FAA, TVSymp, derived from the VFCDM method, exhibited such an association. This indicates that the VFCDM technique may provide additional information not captured by conventional time or time–frequency domain features of the phasic component, such as its mean and standard deviation. Future work could explore the independent contributions of both types of features to better understand their roles in characterizing physiological states.

In relation to the sample size limitations, given the demanding nature of the 25 h sleep deprivation protocol for both researchers and participants, combined with the difficulty of recruiting individuals willing to undergo such an extended period of sleep loss, this study was necessarily conducted with a limited sample of 10 young, healthy subjects. Consequently, the small sample size and the specific demographic of the participants mean that our findings are specific to this group. Therefore, broader generalizations will require additional data collection.

## 6. Conclusions

This study analyzed the EDA (electrodermal activity) and the EEG (electroencephalography) signals that were taken from ten participants developing four cognitive tasks (EAT, ship search, PVT, and N-Back) over 12 trials developed in 24 h. We have found interrelations between EDA features, measured in time and frequency domains, with FAA by means of the mutual information. Such interrelation allowed the identification, by means of the EDA signal, of two main clusters related to high and low FAA values, which can be consequently associated to the positive or negative emotional states of individuals. Additionally, using the support vector machine (SVM) algorithm, we created a model to predict these states for the different cognitive tasks and under deprivation conditions, obtaining good performance in terms of accuracy when a leave-one-trial-out cross-validation strategy is used for robust evaluation. Our findings establish EDA as a promising alternative to FAA for physiological emotion analysis. 

## Figures and Tables

**Table 1:** Pearson’s correlations between frontal alpha asymmetry (FAA) and EDA features for random individuals in Trials 1, 7 and 10, and the corresponding *p*-values.

Trial	Individual	EDA Feature	Pearson’s r	*p*-Value
1	1	SCR_Amp	−0.165	0.03
1	10	SCR_Rise_T	−0.58	0.00019
1	9	TVSymp	−0.65	0.00019
1	9	SCR_Rise_T	−0.84	0.00019
1	10	TVSymp	0.38	0.00019
7	1	Std_Tn	0.0064	0.936
7	8	Std_Tn	−0.382	0.00019
7	2	Mean_Tn	0.76	0.00019
7	5	Mean_CW_Tn	−0.519	0.00019
7	5	SCR_Amp	−0.286	0.00019
10	4	Mean_Tn	0.3	0.00019
10	5	Mean_Tn	0.916	0.00019
10	6	Mean_CW_Tn	−0.532	0.00019
10	1	SCR_Rise_T	0.655	0.00019
10	8	Std_Tn	0.613	0.00019

**Table 2:** Results of the leave one-trial out cross-validation scheme. Using 11 trials for training and 1 for testing across all 12 combinations, this table presents the coincidence rate between class labels (Class 1/Class 2) and the corresponding positive/negative values of the normalized ratio FAA (P_FAA), for all the cognitive tasks. General average performance was approximately 90% for EAT and N-Back tasks, which demand high memory and alertness conditions, and 80% for Ship Search and PVT tasks, related to spatial temporal and vigilance awareness.

Val. Trial	EAT		Ship Search		N-Back		PVT	
	Class 1	Class 2	Class 1	Class 2	Class 1	Class 2	Class 1	Class 2
1	97.9	97.7	74.1	75.3	100	100	71.2	72
2	97.8	97.8	78.1	85.3	100	100	91.3	90.6
3	84.2	83.5	53.3	55.2	70.8	58.8	86.7	89.3
4	95.2	96.1	92	89.1	94.1	94.4	69.2	75.6
5	92.7	94.3	72.5	54.5	88.2	89.5	91.2	90.8
6	94.9	96.3	100	100	87.5	90	91.7	90.2
7	82.1	80	61.8	74.8	100	100	90.3	91.5
8	98.8	99	77.4	88.5	100	100	88.9	92.4
9	83.2	84.5	100	100	88.9	88.9	87.1	86.4
10	88	89.1	86.3	85.5	95	93.3	76.2	81
11	72.4	75.7	94.4	94.8	85	82.4	83	90.9
12	92.6	92.6	78.1	81.8	89.5	88.2	58.2	41.4
**Average**	**90.7%**	**91.3%**	**80.0%**	**82.4%**	**92.5%**	**90.6%**	**80.8%**	**81.7%**

## Data Availability

The raw data supporting the conclusions of this article will be made available by the authors on request.
